# *Eutrema salsugineum* (Cruciferae) new to Mexico: a surprising generic record for the flora of Middle America

**DOI:** 10.3897/phytokeys.76.9731

**Published:** 2017-01-05

**Authors:** Dmitry A. German, Marcus A. Koch

**Affiliations:** 1 Department of Biodiversity and Plant Systematics, Centre for Organismal Studies (COS) Heidelberg, Heidelberg University, Im Neuenheimer Feld 345, D-69120 Heidelberg, Germany; 2 South-Siberian Botanical Garden, Altai State University, Lenin Str. 61, 656049 Barnaul, Russia

**Keywords:** Brassicaceae, floristic finding, geographic disjunction, halophyte, model species, native distribution range, Thellungiella

## Abstract

The paper reports *Eutrema salsugineum* as a novelty to the flora of Mexico and Middle America in general. The finding stands ca. 1600 km apart from the closest known locality in the Rocky Mountains of Colorado, USA. The species is considered native to NW Mexico and its late discovery in the region is presumably explained by its tiny habit, early flowering time, and subephemeral life cycle. The phylogenetic position of this Mexican population in a haplotype network based on the chloroplast DNA fragment *psb*A-*trn*H confirms this hypothesis and also suggests, in contrast to the previously held viewpoint, multiple colonizations of North American continent from Asia.

## Introduction

*Eutrema salsugineum* (Pall.) Al-Shehbaz & Warwick, long known as *Thellungiella salsuginea* (Pall.) O.E. Schulz, is, along with *Eutrema edwardsii* R. Br., the most widely distributed representative of the genus *Eutrema* R. Br., occuring in temperate and, to a lesser extent, the Arctic zone of Eurasia and North America. Being an obligatory halophytic annual related to *Arabidopsis thaliana* (L.) Heynh., it has become a new model for studying abiotic stress (salt, cold, and draught) tolerance in plants (Bressan et al. 2001; Amtmann 2009). As a result, *Eutrema salsugineum* became one of the first representatives of Brassicaceae to have its genome sequenced (Wu et al. 2012). Nevertheless, fundamental (e. g., geographic) information on the species is incomplete (Koch and German 2013). Due to the peculiar ecological requirements of the species, *Eutrema salsugineum* has a highly disjunct distribution in both continents (Korobkov 1975; Ovchinnikova 1994; Rollins 1993; Al-Shehbaz 2010). Despite being described in the 18th century (Pallas 1773) and being noted for its geographic disposition (Schulz 1924), the exact distribution of this species remained unknown, until recently. One of the reasons for the uncertainty was the misidentification of multiple specimens from Russia and Kazakhstan incorrectly referred to as “*Thellungiella salsuginea*”. Of these, all specimens from the western-most part of the range belong to another species, *Eutrema botschantzevii* (D.A. German) Al-Shehbaz & Warwick [*Thellungiella botschantzevii* D.A. German] (German 2002, 2006, 2008). This finding now means that *Eutrema salsugineum* is completely absent from Europe being bounded at the west by the lower reaches of Amu Darya river, Aral Sea, and the Turgai Valley. Our knowledge on the distribution of the species in America had also undergone improvements, and nowadays *Eutrema salsugineum*’s range is known to extend discontinuously from boreal and Arctic Canada (Alberta, Manitoba, Northwest Territories, Saskatchewan, Yukon) down through the Rocky Mountains within British Columbia, and south to Montana and Colorado. Previous reports for other states such as Idaho (Rydberg 1917) and Ontario (Scoggan 1957) were not confirmed by subsequent studies (Al-Shehbaz 2010; Brouillet et al. 2010). Here we report of *Eutrema salsugineum* specimens collected by the second author in 2010 from arid regions in north-eastern Mexico, revealing another highly disjunct and we believe indigenous population. Our findings are supported with additional genetic analyses from which we draw further biogeographic conclusions.

## Materials and methods

The specimen documenting the occurrence of *Eutrema salsugineum* in Middle America is deposited in HEID. For estimation of the newly revealed disjunction in the distribution area of the species, available information regarding the closest occurence has been used (Weber 1966). Results were interpreted in light of the most recent phylogeographic study of *Eutrema salsugineum* by Wang et al. (2015). In this study various plastid genes (*ndh*F1, *ndh*F2, *psb*A-*trn*H, *rpo*C1, *rbc*L, *trn*D-*trn*T, *trn*L, *trn*L-*trn*F, *trn*V) have been sequenced and DNA polymorphisms have been detected in one marker only. In order to find the inter-species affinity of the Mexican population, its position in the haplotype network based on the respective variable chloroplast DNA fragment, *psb*A-*trn*H, was determined. For this purpose, representatives of the two geographically closest populations (from Montana and Colorado) not studied molecularly before and one accession from Canada, as internal control, were also sequenced.

Total DNA was extracted from 100 mg of herbarium tissue using the Invisorb Spin Plant Mini Kit (Stratec Biomedical AG, Birkenfeld, Germany). PCR amplification was performed in a volume of 25 μL, using 10 μM of each primer, respectively, a total of 2.0 mM MgCl_2_ and 0.5 U of Mango-Taq polymerase (Bioline, Luckenwalde, Germany). The primers used for amplification were *psb*A-for: 5'-GTT ATG CAT GAA CGT AAT GCT C-3', and *trn*H-rev: 5'-CGC GCA TGG TGG ATT CAC AAT CC-3'). All primers were extended by the M13 sequence for subsequent sequencing using M13 universal sequencing primers. The amplifications were run on a PTC 200 Peltier Thermal Cycler (MJ Research, Waltham, Massachusetts, USA) under the following conditions: 3 min initial denaturation at 95°C; 30 cycles of amplification with 30s at 95°C, 30s at 50°C, and 1 min at 72°C; and 5 min of final elongation at 72°C. PCR success was checked with electrophoresis in a 1% agarose gel in TAE-buffer. PCR product clean-up was executed using the Wizard SV Gel and PCR Clean-Up System (Promega, Madison, USA). Custom Sanger-sequencing was performed with GATC- Biotech (Konstanz, Germany). The electropherograms were checked and trimmed to the borders of the analysed markers using the program SeqMan DNA-Star Lasergene software package (DNASTAR, Madison, Wisconsin, USA).

DNA sequence variation was compared with cpDNA haplotypes detected by Wang et al. (2015). Haplotype network analysis was conducted with SplitsTree4 vers. 4.14.4 (Huson and Bryant 2006) with gaps treated as additional binary character.

Apart from the Mexican one, the newly sequenced specimens are: 1) USA, Colorado, Park Co., 11 August 1965. *W.A. Weber 12925* (GH); 2) USA, Montana, Beaverhead Co., 20 June 1920. *E.B. Payson & L.B. Payson 1730* (GH); 3) Canada, Saskatchewan, Jameson, 15 miles south of Regina, 15 June 1983. *G.F. Ledingham 7937* (MO).

## Results and discussion

***Eutrema salsugineum*** (Pall.) Al-Shehbaz & Warwick, Harvard Pap. Bot. 10(2): 134. 2005. Described from: [NE Kazakhstan], …circa lacus et lacunas sale praesertim amaro abundantes ad Irtin inter fortalitia Shelesenka et Jamyschewa. Lectotype (designated by German 2011: 52): [Kazakhstan, Pavlodar province, Irtysh valley], “*Sisymbrium salsuginosum*. [29 May 1771, *P.S. Pallas s.n.*], Herb. Pallas. Herb. Fischer” (LE!) ≡ *Sisymbrium salsugineum* Pall., Reise Russ. Reichs 2: 466, 740, tab. V. 1773. ≡ *Arabidopsis salsuginea* (Pall.) N. Busch, Fl. Sibir. Orient. Extr. 1: 136. 1913. ≡ *Thellungiella salsuginea* (Pall.) O.E. Schulz in Engler, Pflanzenreich 86 (4, 105): 252. 1924.

As taxonomy/nomenclature is not the focus of the present study, only most frequently used synonyms are given here. For detailed synonymy, Schulz (1924: 252) and Tropicos (tropicos.org) can be consulted.

New locality: “Mexico: Est. Nuevo León, Los Enebros, Cerro el Potosi, Sierra Madre Oriental, saline-sodic soils, limestone. 24°52'35"N, 100°23'38"W, 1882 m a.s.l., 14 March 2010. *Marcus Koch s.n.*” (HEID 501412: http://gartenbank.cos.uni-heidelberg.de/img/HEID501412).

**Figure 1.**
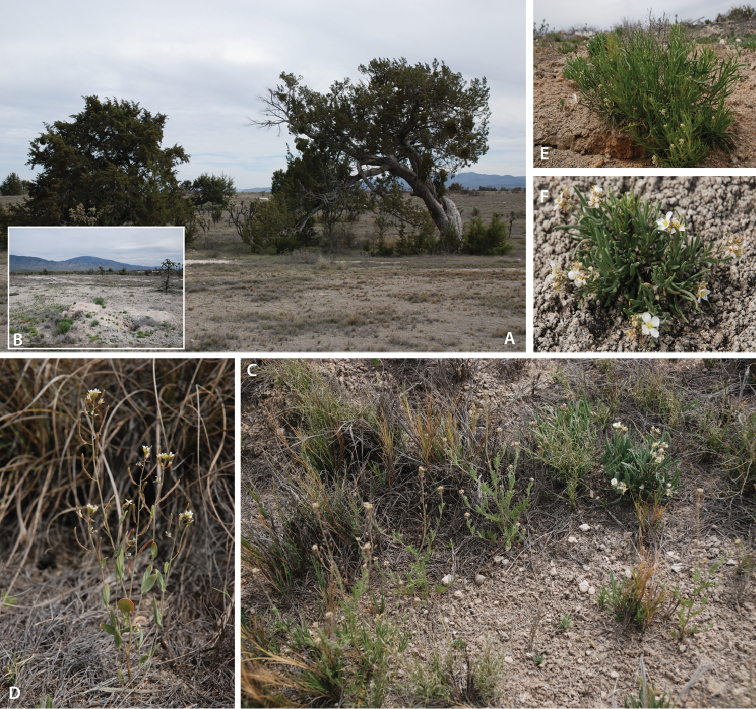
Habitat of Mexican accession of *Eutrema salsugineum* (Los Enebros, Cerro el Potosi, Sierra Madre Oriental). **A, B** General view **C–E** Closer look of *Eutrema salsugineum* and co-occuring Brassicaceae species with herbarium numbers of respective specimens: **C** *Descurainia pinnata* (Walter) Britton s. l. (HEID 501409 to 501411) **D** *Eutrema salsugineum* (HEID 501412) **E** *Lepidium alyssoides* A. Gray (HEID 501414, 501415) **F** *Nerisyrenia linearifolia* (S. Watson) Greene (HEID 501413). All images were taken on March 14^th^ 2010 by M.A. Koch.

This is the first record of *Eutrema salsugineum* (and the genus) from Middle America (specifically Mexico) which shifts the southern limit of the distribution of the species ca. 1600 km to south-southeast from the closest, isolated fragment of the distribution area of the species, confined to Park County, Colorado (Weber 1966). This introduces a hitherto unrecognized disjunction within the New World part of *Eutrema salsugineum*’s range. In Sierra Madre Oriental the plants are found at elevations of ca. 1880 m a.s.l. in a semi-open habitat among halophytic, oak/*Juniperus* (*Juniperus flaccida*)- and grasses-dominated vegetation on alkaline soil which fits well the ecological requirements of the species (Fig. 1). The habitat at the Mexican location of *Eutrema salsugineum* is apparently natural and shows no obvious signs of anthropogenic modification. Having in mind the naturally disjunct biogeography of this species we consider it as a natural element of the flora of Sierra Madre Oriental which was overlooked by previous studies due to its tiny habit, trivial appearance, early flowering time, and short, almost ephemeral life cycle. Results of the haplotype analysis (below) apparently confirm this conclusion.

**Figure 2.**
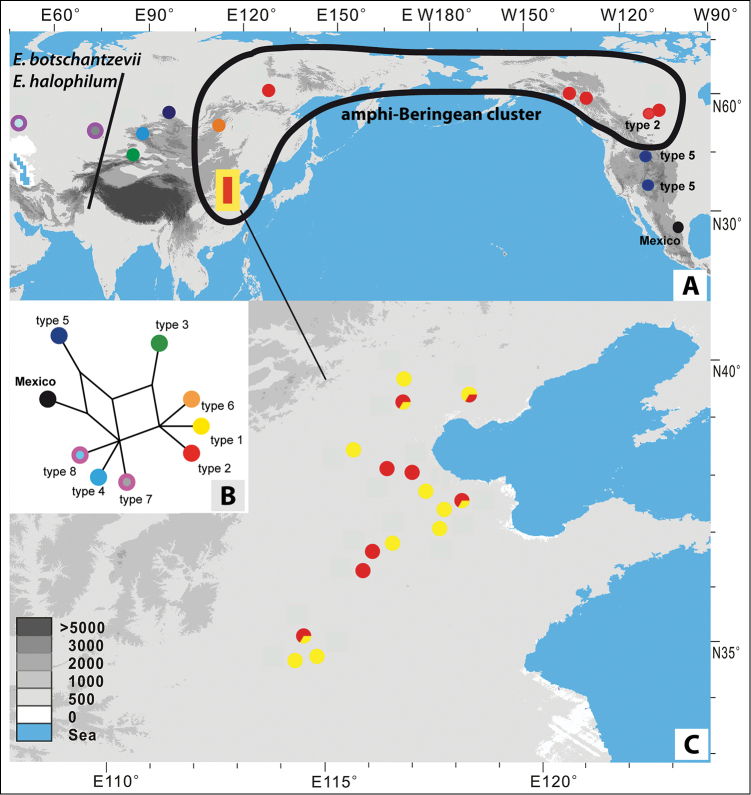
Distribution and relationships of *Eutrema salsugineum* haplotypes. **A** distribution of accessions analyzed herein and in an earlier study (modified from Wang et al. 2015) **B** SplitsTree graph showing genetic relationships among the nine haplotypes (Median-joining network: Bandelt et al. 1999) **C** detailed distribution of haplotypes H1 and H2 in North-West China (modified from Wang et al. 2015).

DNA sequence data revealed a new plastid DNA haplotype of Mexican *Eutrema salsugineum* indicating its genetic distinctiveness. The haplotype code is indicated in Table 1 summarizing also haplotypes detected earlier (Wang et al. 2015). The geographic distribution of the various accessions and their plastid haplotypes are given in Fig. 2. SplitTree analysis indicates that the Mexican plastid haplotype is most closely related to haplotype H5 (numbering following Wang et al. 2015), which was originally found only once in Tuva (South Central Siberia) (Fig. 2; Wang et al. 2015). However, the same H5 haplotype was found here in Montana and Colorado, largely representing the mountain regions of the western United States. As expected, the Canadian sample from Saskatchewan analyzed herein carries haplotype H2, which is widely distributed in NE China and the amphi-Beringian region (Fig. 2c).

**Table 1.** Variable sites of the polymorphic cpDNA fragment *psb*A-*trn*H in *Eutrema salsugineum* and closely related species (modified from Wang et al. 2015). GenBank code of the Mexican haplotype is KY073435.

Species	Haplotype	*psb*A-*trn*H							
		43	108	129	156	162	208	277	304
*Eutrema salsugineum*	H1	A	C	TGAATTT	A	TTTCTAT	A	-	C
	H2	A	C	TGAATTT	A	TTTCTAT	A	-	A
	H3	A	C	TGAATTT	A	ATAGAAA	A	-	A
	H4	A	C	TGAATTT	A	TTTCTAT	C	A	A
	H5	A	C	AAATTCA	G	ATAGAAA	A	A	A
	H6	A	T	TGAATTT	A	TTTCTAT	A	-	A
	Mexican	A	C	AAATTCA	A	TTTCTAT	A	A	A
*Eutrema halophilum*	H7	A	C	TGAATTT	A	TTTCTAT	A	A	A
*Eutrema botschantzevii*	H8	G	C	TGAATTT	A	TTTCTAT	A	A	A

In their recent phylogeographic study Wang et al. (2015) discovered extremely low level of genetic diversity throughout the whole distribution area of *Eutrema salsugineum*, especially in NE Asia/America, apparently reflecting a very recent (ultimately Late-Pleistocene/very Early Holocene) range expansion. These authors suggested wind to be the main agent mediating the fast and long-distance dispersal of *Eutrema salsugineum* and a single colonization of the New World by the species (Wang et al. 2015). However, a present-day fragmentary sublongitudinal distribution of *Eutrema salsugineum* in the Rocky Mountains along with our finding that relevant populations carry a haplotype (H5) which is identical to a unique haplotype found in South Siberia, suggests that the North American continent has been colonized independently two times. This conclusion is supported by the fact that the newly described accession occurring in Mexico carries a haplotype which most likely derived directly from haplotype H5. This overlapping distribution pattern of an amphi-Beringian group carrying H1/H2 and a disjunct group (South Siberia versus western North America) carrying H5 plus the Mexican type can also be best explained by a first and older immigration of *Eutrema salsugineum* from Asia predating the last glaciation. During the Last Glaciation Maximum the northern populations in America apparently went extinct and/or migrated southwards. If this scenario is correct, the last latitudinal shift of vegetation belts could enable the species to reach the Middle Americas and subsequently survive there in appropriate habitats at higher elevation. The wide amphi-Beringian distribution of H1 and H2 might reflect postglacial expansion as demonstrated by the phylogeography inferred by Wang et al. (2015) based on nuclear genes. This multiple immigration pattern of various temperate-cold and often draught adapted Brassicaceae taxa between East Asia and the North America has been documented e.g. also for *Arabidopsis* and *Arabis* L. (Schmickl et al. 2008, 2010; Koch et al. 2010; Koch and Grosser 2017), highlighting the general significance of this spatio-temporal pattern.

## Conclusion

As shown herein, Asian-North American halophyte *Eutrema salsugineum* has a far more southern distribution in the New World than previously considered almost reaching the Northern Tropic. The character of the discovered habitat suggests native rather than human-mediated occurrence of the species in Mexico, a conclusion supported by molecular footprints. A *psb*A-*trn*H-based haplotype network demonstrates more complicated infraspecific structure and biogeographic history of *Eutrema salsugineum* than previously thought and suggests its multiple invasions to the New World.
